# *RET* fusion partners dictate oncogenic potential in undifferentiated spindle cell sarcomas

**DOI:** 10.1080/15384047.2026.2683190

**Published:** 2026-06-04

**Authors:** Qi Gui, Ying Zhang, Mei Yang, Rongrui Liang, Man Huang, Xiaoshan Yang, Nan Chen, Xiaojun Chen, Mengyao Wu, Huafei Chen, Lijun Meng, Sheng Xiao, Min Tao

**Affiliations:** a Department of Oncology, The Fourth Affiliated Hospital of Soochow University, Suzhou, China; b Advanced Molecular Pathology Institute of Soochow University and SANO, Suzhou, China; c Suzhou SANO Precision Medicine Ltd, SANO Medical Laboratories, Suzhou, China, Suzhou, China; d Department of Oncology, The First Affiliated Hospital of Soochow University, Suzhou, China; e Department of Pathology, Brigham and Women's Hospital, Harvard Medical School, Boston, MA, USA

**Keywords:** *RET* fusion heterogeneity, undifferentiated spindle cell sarcoma, *MYH10::RET*, *NTRK1* fusion resistance, sequential targeted therapy, kinase activity disparity

## Abstract

**Background:**

Spindle cell tumors with *RET* rearrangements exhibit variable clinical behaviors, ranging from benign to highly aggressive malignancies. The underlying heterogeneity is suspected to be associated with distinct fusion partner genes. Understanding the impact of these fusion partners on oncogenic potential is crucial for precision therapy.

**Methods:**

We report a case of a spindle cell tumor harboring an *MYH10::RET* fusion, which initially responded to anti-RET therapy but relapsed due to an *NTRK1* fusion. Isogenic cell lines expressing MYH10::RET and CCDC6::RET were established. Functional assays, including cell proliferation, migration, invasion, and kinase activity assays, were performed. Genomic profiling was conducted using targeted DNA and RNA NGS and FISH.

**Results:**

MYH10::RET-expressing cells showed significantly higher proliferation, migration, and invasion compared to CCDC6::RET-expressing cells. MYH10::RET exhibited approximately three-fold higher kinase activity. The patient's disease was managed through sequential targeted therapies, including combination therapy with third-generation inhibitors targeting RET and NTRK.

**Conclusion:**

Our findings suggest that distinct *RET* fusion partners significantly contribute to the clinical heterogeneity of *RET*-rearranged spindle cell tumors, likely through differential kinase activity. Continuous genomic monitoring is essential for identifying resistance mechanisms and guiding precision therapy. Future studies should explore the impact of different fusion partners on tumor behavior and therapeutic response.

## 1. Introduction

Soft tissue tumors are characterized by the activation of various tyrosine kinases, mostly through chromosome translocations, resulting in the formation of chimeric proteins, along with activation point mutations within the tyrosine kinase domains or insertions/deletions within the juxtamembrane domains. To date, 10 tyrosine kinases have been identified in this group of tumors: *RET*, *NTRK1/2/3*, *ROS1*, *ALK*, *EGFR*, *MET*, *BRAF*, and *RAF1.* ^1-3^

Among these alterations, *NTRK* fusions are among the best characterized kinase rearrangements in soft tissue tumors. The canonical *ETV6::NTRK3* fusion is detected in over 90% of infantile fibrosarcomas, but remains rare in adult soft tissue sarcomas, with a reported prevalence of <1–3%.^4^ ^,^ ^5^ A large systematic review encompassing 18,077 sarcoma patients further estimated an overall *NTRK* fusion frequency of 3.05%.^6^ In contrast, *RET* fusions are distinctly uncommon in soft tissue tumors.^7^ However, genomic studies have identified *RET* rearrangements in a subset of spindle cell soft tissue tumors with morphological overlap with *NTRK*-rearranged neoplasms, suggesting that these tumors may belong to a broader spectrum of kinase-fusion–driven sarcomas.^8^ *RET* fusions are established oncogenic drivers in several epithelial malignancies, particularly papillary thyroid carcinoma and nonsmall cell lung cancer, occurring in approximately 5–10% and 1–2% of cases,^9-11^ respectively. Selective RET inhibitors, such as selpercatinib and pralsetinib, have shown substantial clinical efficacy. Oncogenic kinase fusions are generally mutually exclusive driver events, and the secondary acquisition of alternative kinase fusions, such as *NTRK* rearrangements, during RET-targeted therapy remains rare, with only isolated cases in RET-driven lung and thyroid cancers.^12^ ^,^ ^13^

Spindle cell tumors with *RET* rearrangements exhibit diverse histopathologic characteristics, similar to those observed in lipofibromatosis-like neural tumors (LPF-NT), infantile fibrosarcomas (IF), malignant peripheral nerve sheath tumors (MPNST), and fibrosarcoma. While some of these tumors express S100 and CD34, their immunophenotypes are generally nonspecific.^8^ Emerging evidence suggests that specific *RET* fusion partners may be associated with distinct clinicopathologic phenotypes.^14^ *CCDC6::RET* and *NCOA4::RET* rearrangements have been reported in tumors resembling lipofibromatosis-like neural tumor (LPF-NT), which are typically indolent and managed primarily by surgical resection in both soft-tissue and epithelial settings.^3^ In contrast, other *RET* fusions, such as *MYH10::RET*, have been identified in tumors with high-grade histologic morphology and aggressive clinical courses, including metastatic disease.^14^

The biological basis underlying these distinct clinical behaviors remains unclear. Even though oncogenic receptor tyrosine kinases (RTKs) activate comparable downstream signaling pathways, the distinct fusion partners of RTKs can modulate the behavior of tumor cells. For example, NIH3T3 cells expressing seven *ALK* fusions containing various 5'-fusion partners demonstrated different cell growth in soft agar cultures and varying sensitivities to *ALK* inhibitors. Additionally, *ALK* fusion proteins exhibit different kinase activities.^15^ Fusion partners can also affect cellular localization; For instance, *ETV6::NTRK3* expressed in melanocytes was found in both the nucleus and cytoplasm, leading to epithelioid morphology, whereas *MYO5A::NTRK3* was exclusively nuclear and resulted in spindle cell morphology.^16^ Similar variability was observed for the non-RTK fusion proteins. In acute promyelocytic leukemia (APML), patients with the classic *PML::RARA* fusion respond to all-trans retinoic acid (ATRA), resulting in a cure for once-deadly leukemia. Conversely, patients with nonclassic *RARA* fusions featuring different 5' partners show varied responses to ATRA, with some being unresponsive to therapy.^17^

In this study, we present a case of undifferentiated spindle cell sarcoma harboring the *MYH10::RET* fusion, which exhibited a fibrosarcoma-like morphology and lung metastasis. To investigate why different *RET* fusions led to varying clinical outcomes, we established isogenic cell lines stably expressing either MYH10::RET or CCDC6::RET. We found that cells expressing MYH10::RET exhibited increased proliferation, migration, and invasion compared to those expressing CCDC6::RET, thereby reflecting their distinct clinical behaviors. Further analysis showed that MYH10::RET had a stronger kinase activity than CCDC6::RET. We concluded that the activation level of RET is modulated by different fusion partners, likely contributing to the heterogeneous clinical behaviors observed in these tumors. These results may have clinical implications and may potentially influence patient management strategies.

## 2. Materials and methods

A preprint version of this manuscript has been posted on Research Square.^18^ The tumor tissue analyzed in this study was derived from a lung needle biopsy specimen obtained from the patient. The biopsy sample was routinely processed as formalin-fixed and paraffin-embedded (FFPE) for histopathological evaluation and subsequent molecular analyzes.

### 2.1. Immunohistochemistry

FFPE tumor tissue sections were cut at a thickness of 5 μm for immunohistochemical analysis.^19^ ^,^ ^20^ Immunohistochemical staining for pan-TRK (ab181560, Abcam, UK), CD34 (Kit-0004, MXB, China), and S100 (ab11428, Abcam, UK) was performed as follows: The slides were baked at 60 °C for 1 h, deparaffinized, and rehydrated through a series of 100% xylene, 100% ethanol, and running water.^20^ ^,^ ^21^ They were then blocked with a solution composed of 10% normal serum (100 µL per slide; Cat: S-1000, Vector Laboratories, USA) and 1% bovine serum albumin (BSA) (100 µL per slide; Cat: A9647, Sigma-Aldrich, USA) in Tris-buffered saline (TBS, pH 7.4; 100 µL per slide; Cat: T5030, Sigma-Aldrich, USA), followed by incubation with primary antibodies for 2 h.^22^ Endogenous peroxidase activity was quenched using a 0.3% hydrogen peroxide solution (100 µL per slide; Cat: H1009, Sigma-Aldrich, USA), and the slides were subsequently incubated with a horseradish peroxidase (HRP)-conjugated polymer detection system (EnVision™ Detection System; 100 µL per slide; Cat: K5007, DAKO, Agilent Technologies, Denmark).^22^ The tissue sections were visualized with 3,3′-diaminobenzidine (DAB) chromogen (100 µL per slide; Cat: K3468, DAKO, Agilent Technologies, Denmark) as the chromogen and counterstained with Mayer's hematoxylin (approximately 100 µL per slide; Cat: MHS16, Sigma-Aldrich, USA).^21^

### 2.2. Targeted DNA next-generation sequencing (NGS)

Genomic DNA was extracted from FFPE tumor tissue sections using the QIAamp DNA Micro Kit (Cat: 56,304; Qiagen, Germany).^23^ DNA samples (300 ng) were fragmented to approximately 200–300 bp using the Bioruptor Pico (Diagenode, Denville, NJ, USA). Library preparation was conducted with the Rapid Plus DNA Lib Prep Kit for IlluminaRK (20208, ABclonal, China) following the manufacturer's protocol.^24^ ^,^ ^25^ The libraries were hybridized with a pool of biotinylated bait oligonucleotides targeting 638 cancer-related genes for 16 h.^25^ The targeted sequences were captured using streptavidin-coated beads, and then amplified by PCR.^26^ The samples were sequenced as paired-end 150-bp reads on the Illumina NextSeq 6000 platform.^26^ The sequencing data were aligned to the human reference genome (hg19) using BWA-MEM (RRID: SCR_010910).^26^ Variants, including single-nucleotide variations (SNVs), insertions/deletions (indels), copy number variations (CNVs), and structural variations (SVs), were analyzed using SeqNext software (JSI, Germany) and custom pipelines developed by Sano Medical Laboratories (China).^26^

### 2.3. Fluorescence in situ hybridization (FISH) analysis

FISH analysis was conducted on 5-μm FFPE tumor tissue sections using dual-color split-apart probes (5' probe labeled green and 3' probe labeled red) for RET and NTRK1, sourced from Betrue, China.^26^ The slides were deparaffinized using xylene, rehydrated, and subjected to digestion with a 750 U/ml pepsin solution (Cat: P6887, Sigma Aldrich, USA) for 10 minutes, followed by incubation in 10% buffered formalin (Cat: E672001-0100, Sangon Biotech, China; ~40–50 mL per Coplin jar) for an additional 10 minutes.^27^ Both the slides and probes were denatured separately at 95 °C, and hybridization was carried out overnight at 37 °C.^27^ Post-hybridization, the slides were washed in 0.4 × saline–sodium citrate buffer (SSC) containing 0.3% NP-40 (approximately 50 mL per Coplin jar; SSC stock solution Cat: S6639, Sigma-Aldrich, USA; NP-40, IGEPAL CA-630; Cat: I8896, Sigma-Aldrich, USA) at 73 °C for 3 minutes and subsequently counterstained with DAPI (Cat: P0131, Beyotime, China).^27^

### 2.4. Targeted RNA NGS

RNA was extracted from FFPE tumor tissue sections using Trizol reagent as per the manufacturer's protocol (Cat: 10296010, ThermoFisher, Invitrogen, USA).^26^ Reverse transcription was carried out with 100 ng of total RNA.^28^ Subsequent end repair and adapter ligation steps were performed in accordance with standard NGS procedures (Cat: E7771 and E6111, NEB, USA).^20^ PCR enrichment was conducted using 641 gene-specific primers targeting 118 genes frequently implicated in solid tumors.^20^ The enriched PCR products were sequenced on the NovaSeq 6000 platform (Illumina, USA), and the sequencing data were analyzed using SeqNext software (JSI, Germany).^20^

### 2.5. Lentivirus infection

The murine fibroblast cell line NIH3T3 (RRID: CVCL_0594) was obtained from the laboratory and maintained under standard culture conditions. Cells were cultured in Dulbecco's modified Eagle's medium (DMEM; Cat: CGM101.05, CellMax, China) supplemented with 10% fetal bovine serum (FBS; Cat: SA211, CellMax, China) and 1% penicillin–streptomycin solution (Cat: P1410, Solarbio, China; final concentration: 100 U/mL penicillin and 100 μg/mL streptomycin) at 37 °C in a humidified incubator with 5% CO. NIH/3T3 cells, a classical transformation model with low background signaling and no relevant driver mutations, were chosen for these experiments to provide a controlled cellular context for assessing the intrinsic transforming potential of the fusion proteins. The *CCDC6::RET* and *MYH10::RET* fusion genes were cloned into the lentiviral plasmid vectors. These plasmids, along with empty vectors for control cells, were cotransfected with psPAX2 and pMD2.G into NIH3T3 cells (RRID: CVCL_0594). The supernatant from transfected NIH3T3 cells was harvested and filtered 48–72 h post-transfection.^29^ Target cells were subsequently infected with lentiviral particles in the presence of 10 μg/mL polybrene (Cat: TR-1003-G, Sigma-Aldrich, USA).^29^ After two weeks of puromycin (Cat: HY-K1057, MedChemExpress (MCE), USA) selection, the cells were collected for further experiments.^29^

### 2.6. Cell counting Kit-8 Assay

The cells were subsequently seeded into 96-well plates.^30^ After cell attachment, the cells were treated with various TKIs for 3 to 4 d, including selpercatinib (HY-114370, MCE, USA), pralsetinib (S8716, Selleck, USA), and cabozantinib (S1119, Selleck, USA). The culture medium was then mixed at a 10:1 ratio with the CCK-8 solution (Cat: C0039, Beyotime, China) and added to the wells in the dark. After 2 h of incubation at 37 °C, the absorbance (OD) at 450 nm was measured using a microplate reader.^31^

### 2.7. Wound healing assay

NIH3T3 cells were harvested and plated on both sides of the scratch chamber (Culture-Insert 2 Well in µ-Dish 35 mm, Cat: 81176, IBIDI, Germany).^32^ The cells were cultured in a medium supplemented with 10% fetal bovine serum and 1% penicillin until they adhered to the chamber wall.^33^ Following the removal of the scratch chamber, the cells were in serum-free medium.^34^ The wound healing rates of the various cell groups were assessed at 0, 24, and 48 h.^35^

### 2.8. Transwell migration assay

The Transwell insert was placed in a 24-well culture plate. NIH3T3 cells were harvested and resuspended in serum-free medium (24-well format, 8 μm pore size; Cat: 3422, Corning, USA). A total of 200 µL of the cell suspension was added to the upper chamber and 600 µL of medium containing 10% serum was added to the lower chamber as a chemoattractant. Following incubation at 37 °C for 48  h, the chamber was gently washed twice with PBS (phosphate-buffered saline; Cat: BL302A, Biosharp, China), fixed with methanol (Cat: P1110, Solarbio, China), and stained with crystal violet solution (Cat: C0121, Beyotime Crystal Violet, China).

### 2.9. Immunofluorescence colocalization

Approximately 5 × 10⁴ cells were seeded in confocal petri dishes. Once the cells had attached, they were fixed with formaldehyde solution and incubated overnight with a mouse anti-FLAG tag antibody (bsm-33346M, Bioss, USA, diluted 1:200).^36^ The next day, the cells were incubated with a fluorescently labeled secondary antibody (ab150115, Abcam, USA, diluted 1:200) for 1 h.^37^ After washing with PBS (Cat: BL302A, Biosharp, China), an antifade solution containing DAPI (Cat: P0131, Beyotime, China) was added. Fluorescence images were then captured using an Olympus fluorescence microscope.^38^

### 2.10. Western blot analysis and co-immunoprecipitation (CO-IP)

Protein concentrations in cell lysates were measured using a BCA Protein Assay Kit (Cat: 23225, Thermo Fisher, Invitrogen, USA).^39^ Equal quantities of protein lysates were resolved by sodium dodecyl sulfate-polyacrylamide gel electrophoresis (SDS-PAGE) and transferred onto polyvinylidene fluoride (PVDF) membranes (IPVH00010, Millipore, USA).^40^ The membranes were blocked with 5% skim milk (36120ES76; Yeasen, China) for 1 h at room temperature, then incubated with primary antibodies overnight at 4 °C with shaking.^40^ After washing three times with TBST buffer (T1082, Solarbio, China), the membranes were incubated with anti-rabbit or anti-mouse secondary antibodies for 1 h at room temperature and visualized using an enhanced chemiluminescence detection solution (Cat:12003154, Bio-Rad, USA).^40^ ^,^ ^41^ For immunoprecipitation, the protein lysates were incubated with an Anti-Flag Tag and microbeads (A36797, Thermo Fisher, USA), and the free proteins in the supernatant were removed by magnetic separation. Proteins were eluted from the beads and analyzed by western blotting. The antibodies used were as follows: Phospho-RET (Tyr905) antibody (Cat: 3221, CST, USA, RRID: AB_2179887), RET (E1N8X) XP® rabbit mAb (Cat: 14556, CST, USA, RRID: AB_2798509), mouse anti-Flag tag antibody (bsm-33346M, Bioss, USA, 1:200, RRID: AB_3083063), and rabbit anti-beta-actin (Loading Control) antibody (BS-0061R, BIOSS, USA, RRID: AB_10855480).

### 2.11. Kinase activity assays

The activities of the two *RET* fusion kinases were assessed using the ADP-Glo Kinase Assay Kit (Promega Corporation, Madison, WI, USA).^42^ The reaction reagents were prepared in accordance with the manufacturer's protocol.^43^ In each reaction setup, CO-IP-purified RET kinase samples, a synthetic tyrosine-containing peptide substrate (1 mg/mL), and different concentrations of ATP (ranging from 0 to 1000 μM) were added. The mixture was then incubated at room temperature for 60 minutes to enable the kinase to catalyze the phosphorylation of the substrate.^44^ ADP-Glo™ Reagent (final concentration 10 μM) was added to each well and gently shaken to ensure mixing to terminate the kinase reaction. The wells were kept at room temperature for 40 minutes to facilitate the conversion of ADP to ATP.^45^ Subsequently, Kinase Detection Reagent was added to each well, mixed gently, and incubated at room temperature for an additional 60 minutes.^46^ The luminescence signals were then measured using a chemiluminescence detector.^46^

### 2.12. Statistical analysis

All quantitative experiments were performed with at least three independent biological replicates unless otherwise stated. Data are presented as mean ± standard error of the mean (SEM). Statistical analyzes were conducted using GraphPad Prism software (GraphPad Software, San Diego, CA, USA).

Comparisons between two groups were performed using two-tailed unpaired Student's t-tests. Comparisons among multiple groups were conducted using one-way or two-way analysis of variance (ANOVA), followed by Bonferroni post hoc correction where appropriate. For kinase activity assays involving multiple ATP concentrations, two-way ANOVA with Bonferroni correction was applied.

A *p*-value < 0.05 was considered statistically significant. Statistical significance is indicated in the figures as follows: *p* < 0.05 (*), *p* < 0.01 (**), *p* < 0.001 (***), and *p* < 0.0001 (****).

## 3. Results

### 3.1. Genomic profile of a *RET*-rearranged spindle cell tumor

**Figure 1.**
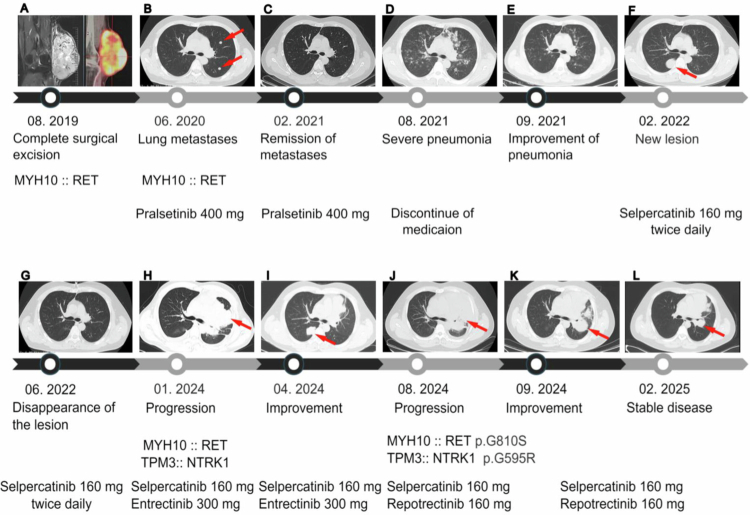
Disease timeline. (A) PET-CT and MRI showed a metabolically active mass on August 20, 2019. The grayscale background represented the anatomical structure. Tumor contained *MYH10::RET*. (B) Multiple metastatic lesions (red arrow) appeared in the left lung on June 10, 2020 (CT lung window). Lung tumor also contained *MYH10::RET*. (C) Significant reduction in lung metastases after Pralsetinib therapy, nearly achieving complete remission, was observed on February 8, 2021 (CT lung window). (D) Severe lung infection was evident on August 12, 2021 (CT lung window). (E) Marked improvement in lung infection was noted on September 2, 2021. (F) A new lesion in the right lung (red arrow) developed on February 15, 2022, six months after discontinuing treatment. (G) Following treatment with Selpercatinib, the lung lesion was nearly resolved by June 10, 2022. (H) Renewed progression of pulmonary lesions with pericardial effusion was observed on January 18, 2024. Biopsy revealed a *TPM3::NTRK1* rearrangement in addition to the *MYH10::RET* fusion. (I) Significant improvement in the left pulmonary lesion and pericardial effusion was noted on April 18, 2024, with combination therapy of Entrectinib and Selpercatinib. (J) Disease progression with recurrent pulmonary lesions and pericardial effusion was observed on August 13, 2024 (CT lung window). Biopsy showed kinase domain mutations from both *TPM3::NTRK1* and *MYH10::RET*. (K) Tumor shrinkage was significant on September 20, 2024, with combination therapy of Repotrectinib and Selpercatinib. (L) The most recent follow-up on February 20, 2025, showed continued reduction in pulmonary metastases.

**Figure 2.**
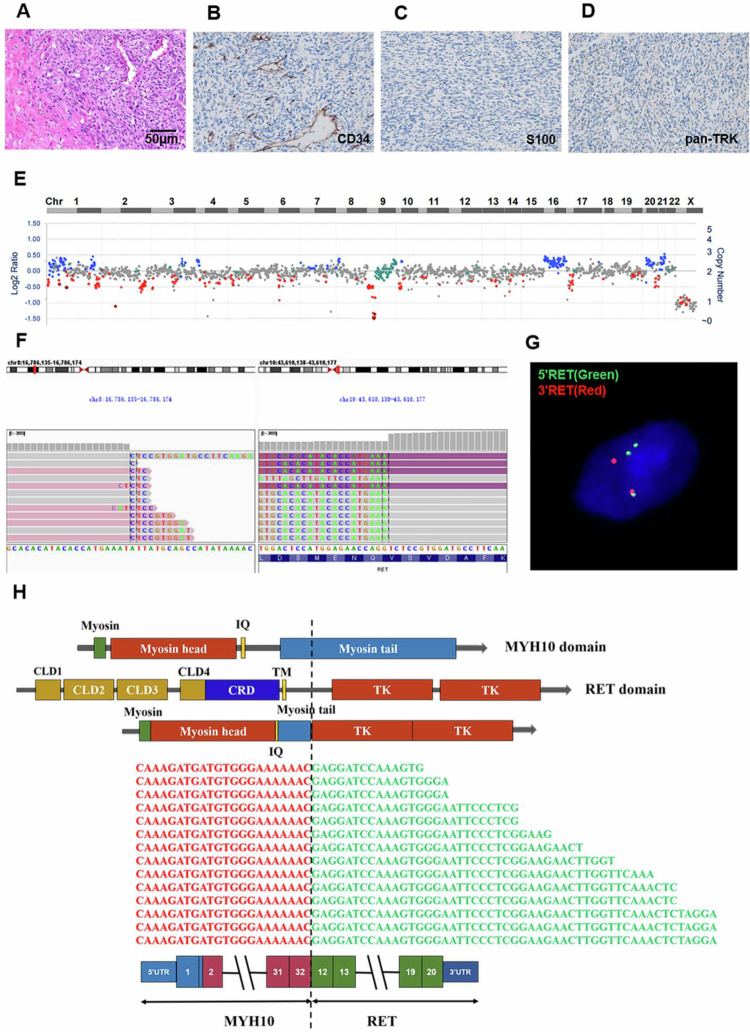
Genomic profiling of the primary spindle cell tumor. (A) H&E staining showed spindle cells arranged in fascicles, with hemangiopericytoma-like vasculature, and areas of necrosis observed. (B–D) IHC was negative for CD34, S100, and pan-TRK. (E) Targeted DNA NGS analysis showed CNVs including *CDKN2A/B* homozygous loss at 9p21 and gain of chromosomes 1p, 16, 20q, and 21. (F) IGV images showed a nonfunctional *RET* rearrangement between *FGF20* and *RET*. (G) FISH showed that the 5′RET green signal separated from the 3′RET red signal, confirmed *RET* rearrangement. Note that an extra 5′RET signal was present, consistent with a complex *RET* rearrangement. (H) Targeted RNA NGS identified the *MYH10::RET* fusion, in which the first 32 exons of *MYH10* are fused with the last 9 exons of *RET*. The exon structure and functional domains of wild-type MYH10, RET, and MYH10::RET fusion are illustrated in a schematic. The breakpoint is indicated by a vertical dotted line. IQ: IQ calmodulin-binding motif; CLD1-4: RET Cadherin-like domain1–4; CRD: Cysteine-rich domain; TM: transmembrane domain; TK: Tyrosine Kinase domain.

A 37-y-old man was admitted with a subcutaneous lump on the outer side of his left knee. PET-CT and MRI revealed a well-defined, hypermetabolic soft tissue mass measuring 159 × 92 × 100 mm, containing both solid and cystic/necrotic components (Figure 1A). A complete surgical excision was performed. Histological analysis of formalin-fixed, paraffin-embedded (FFPE) tissue revealed hypercellular spindle cells arranged in fascicles, with hemangiopericytoma-like vasculature and areas of necrosis (Figure 2A). Immunohistochemical staining revealed a null immunophenotype profile, with tumor cells negative for CD34, S100, and pan-TRK (Figure 2B–D). Based on these findings, a diagnosis of malignant undifferentiated spindle cell tumor with *RET* fusion was confirmed.

Targeted DNA NGS revealed *CDKN2A/B* homozygous loss, 9q copy-neutral loss of heterozygosity (CN-LOH), gain of chromosomes 1p, 16, 20q, and 21, and an *FGF20::RET* rearrangement. The *RET* breakpoint is located in intron 11, which is a common site for *RET* rearrangements. However, due to incompatible transcriptional orientation, the *FGF20::RET* rearrangement did not contain a reading frame (Figure 2E and F). Fluorescence in situ hybridization (FISH) using a RET split-apart probe confirmed the presence of RET rearrangement (Figure 2G). Subsequently, targeted RNA NGS identified an *MYH10::RET* fusion with an intact reading frame and breakpoints, which were consistent with previously reported *MYH10::RET* rearrangements (Figure 2H). The MYH10::RET fusion protein contained the myosin head domain from MYH10 and the intact kinase domain from RET. The oncogenic mechanism likely involves myosin head-mediated dimerization, leading to autophosphorylation and RET kinase activation. The discrepant results between DNA and RNA NGS are likely due to multiple *RET* fusions at the DNA level, including both *FGF20::RET* and *MYH10::RET*. DNA NGS detected nonfunctional *FGF20::RET* but missed functional *MYH10::RET*. This underscores the superiority of RNA NGS over DNA NGS in detecting functional fusions as it effectively eliminates all non-transcript rearrangements often observed in DNA NGS, as previously reported.^47^ ^,^ ^48^

**Figure 3.**
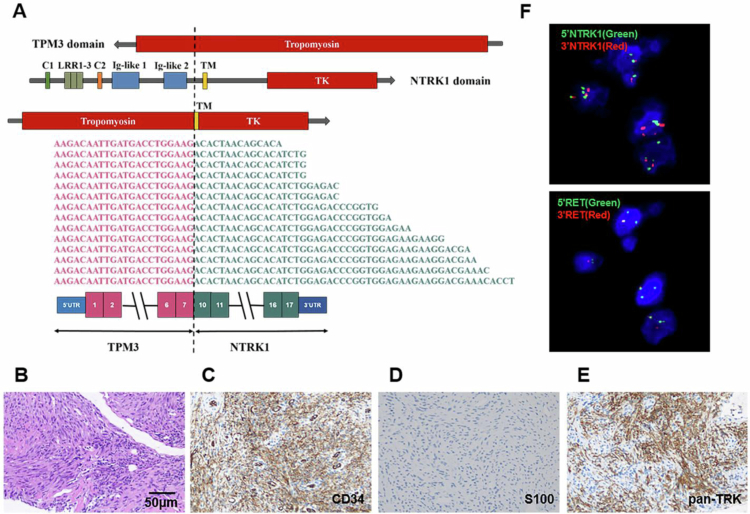
Genomic profiling of the metastatic lesions. (A) Targeted RNA NGS identified a novel *TPM3:NTRK1* fusion, in which exons 1–7 of *TPM3* are fused to exons 12–19 of *NTRK1*. The exon structure and functional domains of wild-type *TPM3*, *NTRK1*, and the *TPM3:NTRK1* fusion are depicted schematically, with the breakpoint indicated by a vertical dotted line. C1-2: Cysteine clusters C1 and C2; LRR1-3: Leucine-rich regions (LRR) 1–3; Ig-like 1-2: Immunoglobulin-like domain 1-2; TM: transmembrane domain; TK: Tyrosine Kinase domain. (B) H&E staining revealed that the lung tumor exhibited similar morphology to the primary knee tumor. (C–E) IHC was positive for CD34 and pan-TRK, and negative for S100. (F) FISH confirmed *NTRK1* rearrangement, showing separation of *5'NTRK1* (green signals) from *3'NTRK1* (red signals). The same slide was stripped and re-hybridized with a *RET* probe set, revealing *RET* rearrangements in the same group of cells, indicated by the separation of 5'*RET* (green signals) from 3'*RET* (red signals).

Ten months after the surgery, the patient developed lung metastasis (Figure 1B), which was biopsied and confirmed to carry the same *MYH10::RET* fusion. The patient was included in a clinical trial and received treatment with 400 mg of pralsetinib (BLU-667). This treatment led to a substantial decrease in lung metastases, nearly achieving complete remission (Figure 1C). However, 11 months later, the patient developed a severe cough and fever. Chest computed tomography (CT) showed severe infection in both lungs and potentially drug-associated pneumonia (Figure 1D). Subsequently, Pralsetinib was discontinued and the patient withdrew from the clinical trial. Microbiome NGS of bronchoalveolar lavage fluid detected human herpesvirus 5 (cytomegalovirus) infection with 45.28% abundance and Pneumocystis infection. Treatment with Ganciclovir, Cotrimoxazole, and human hemoglobin effectively controlled the bilateral lung infections. At this time, chest CT showed no evident tumor lesions (Figure 1E), and the patient did not receive further antitumor therapy but underwent regular follow-up. Six months later, a new lesion had developed in the right lung (Figure 1F). The patient was subsequently treated with 160 mg selpercatinib twice daily, which resulted in significant tumor shrinkage and stable disease (Figure 1G). Nearly 2 y later, the patient presented with marked chest tightness and dyspnea. Chest computed tomography (CT) indicated renewed progression of the pulmonary lesion, accompanied by pericardial effusion (Figure 1H). A lung tumor biopsy was then performed, which showed a new *TPM3::NTRK1* rearrangement in addition to *MYH10::RET* fusion (Figure 3A). Histologically, the tumor cells showed similar morphology to the primary knee tumor, but immunohistochemistry (IHC) showed positivity for CD34 and pan-TRK, which are negative markers in the primary tumor (Figure 3B–E).

To determine whether the same tumor cells harbored both *MYH10::RET* and *TPM3::NTRK1* rearrangements, or if they were present in different cell populations, successive FISH was performed on the same cell group, first with a *RET* split-apart probe set, followed by an *NTRK1* split-apart probe set. This finding demonstrated that both rearrangements coexisted within the same cells (Figure 3F), indicating that the *MYH10::RET* fusion likely functions as a resistance mechanism against the RET inhibitor selpercatinib. The patient was treated with a combination of selpercatinib (160 mg/d) and the *NTRK* inhibitor entrectinib (300 mg/d). This combination significantly shrank the neoplasm in the left lung after two months of treatment (Figure 1I), but a new lesion emerged in the right lung. Over the following 6 months, however, the combination therapy became less effective, and the pulmonary lesions and pericardial effusion showed progression (Figure 1J). Genomic profiling of a tumor biopsy specimen revealed drug-resistant mutations in the kinase domains of both kinases*: RET* p.G810S (variant allele frequency, VAF 1.8%) and *NTRK1* p.G595R (VAF 14.2%). Consequently, the patient transitioned to a combination therapy of selpercatinib 160 mg daily and repotrectinib 160 mg daily, specifically designed to address solvent-front and gatekeeper mutations associated with 1^st^ and 2^nd^ generation therapies. Eleven days later, a chest CT scan revealed significant tumor shrinkage (Figure 1K). With this combination regimen, the patient's disease remained stable, as confirmed by the most recent follow-up (Figure 1L).

### 3.2. Functional evaluation of *MYH10::RET* and *CCDC6::RET*

**Figure 4.**
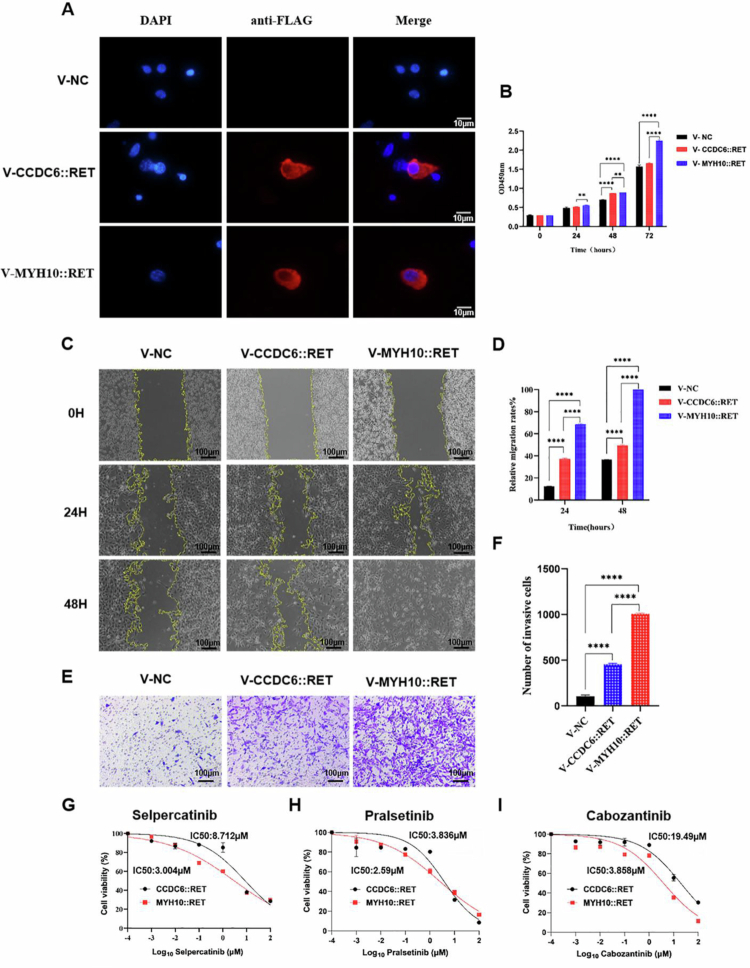
Functional evaluation of MYH10::RET and CCDC6::RET. (A) Both MYH10::RET and CCDC6::RET are located in the cytoplasm of lentivirus-transduced NIH3T3 cells. Cells expressing MYH10::RET exhibited enhanced proliferation (B), migration (C, D), and invasion (E, F) compared to cells expressing CCDC6::RET and control cells with empty vectors. **, *p* < 0.01, ****, *p* < 0.0001. (G-I) Both MYH10::RET-expressing cells and CCDC6::RET-expressing cells were sensitive to the RET inhibitors selpercatinib (G), pralsetinib (H), and cabozantinib (I).

Our patient with *MYH10::RET* had a metastatic tumor characterized by a continuously evolving cancer genome. However, tumors with other *RET* rearrangements, such as *CCDC6::RET*, are often benign. To determine whether distinct RET fusion partners influence oncogenesis at the cellular level, we generated cell lines expressing either *MYH10::RET* or *CCDC6::RET*. Cells were transduced with lentiviruses expressing MYH10::RET or CCDC6::RET, and expression was confirmed by western blotting and immunofluorescence, demonstrating cytoplasmic localization of both fusion proteins (Figure 4A). Cells expressing CCDC6::RET exhibited only a slight increase in growth compared to control cells. In contrast, cells expressing MYH10::RET demonstrated significantly higher proliferation rates than both CCDC6::RET-expressing and control cells at all time points (24, 48, and 72 h), as assessed by the WST-8 assay (CCK-8) (Figure 4B).

In the cell scratch assay, both MYH10::RET*-* and CCDC6::RET-expressing cells showed significantly faster migration than control cells (*p* < 0.0001), with MYH10::RET-expressing cells demonstrating markedly greater migration than CCDC6::RET-expressing cells at 24 h and 48 h post-wounding (Figure 4C and D). In a similar collagen-coated transwell assay to evaluate invasive potential, cells expressing MYH10::RET and CCDC6::RET were both significantly more invasive than control cells (*p* < 0.0001). However, MYH10::RE*T*-expressing cells demonstrated a higher degree of invasiveness compared to CCDC6::RET-expressing cells (*p* < 0.0001) (Figure 4E and F).

Cells expressing CCDC6::RET and MYH10::RET fusions were treated with three tyrosine kinase inhibitors specifically targeting RET. Both cell lines were sensitive to all three inhibitors (Figure 4G–I), although the degree of sensitivity varied. The IC50 values for MYH10::RET-expressing cells were 3.858 μM for cabozantinib, 3.004 μM for selpercatinib, and 2.59 μM for Pralsetinib. In contrast, the IC50 values for CCDC6::RET cells were 19.49 μM for Cabozantinib, 8.712 μM for Selpercatinib, and 3.836 μM for Pralsetinib. These results suggest that MYH10::RET-expressing cells are more sensitive to RET inhibitors than CCDC6::RET-expressing cells, likely reflecting the faster growth of MYH10::RET cells.

**Figure 5.**
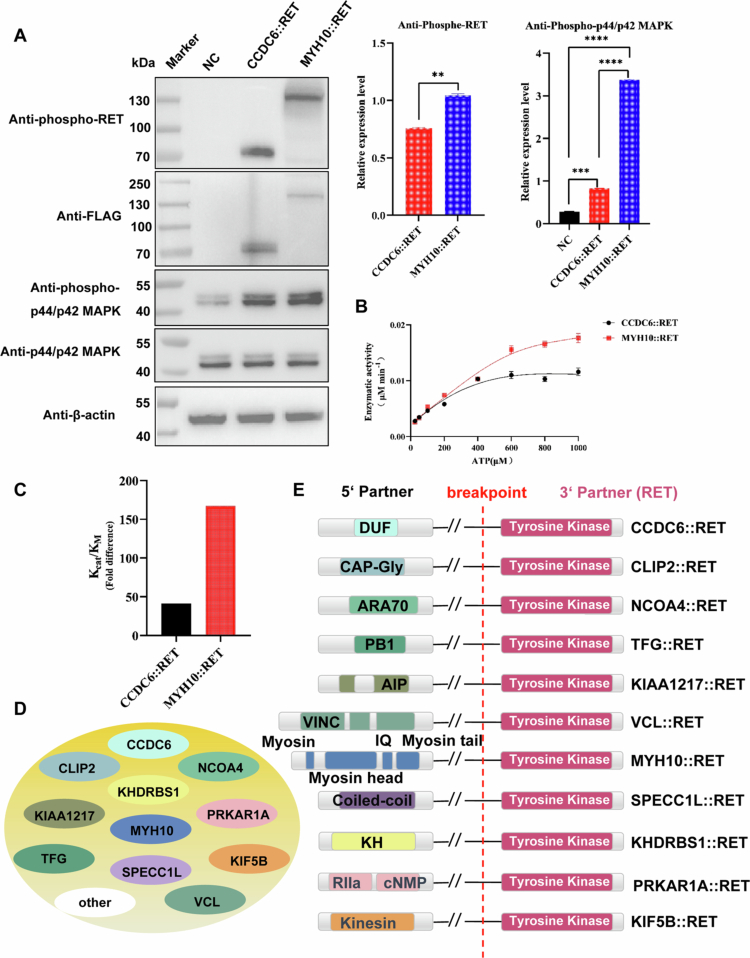
Kinase activities of MYH10::RET and CCDC6::RET. (A) Western blotting showed phosphorylation of both MYH10::RET and CCDC6::RET and their downstream MAPK. The signals were detected using the LiCor Odyssey system, and the levels of phospho-RET, MAPK, and phospho-MAPK were quantified using ImageJ software. **, *p* < 0.01, ***, *p* < 0.001, ****, *p* < 0.0001. (B) MYH10::RET and CCDC6::RET were pulled down from cell lysates, and kinase assays were performed using a synthetic tyrosine-containing peptide substrate (1 mg/mL) with increasing concentrations of ATP. Data are presented as the mean ± SEM, with *n* = 4 from two independent protein preparations; **, *p* < 0.0001, analyzed by two-way ANOVA with Bonferroni correction. (C) Catalytic efficiency constants (Kcat/KM, fold difference). (D) Currently reported *RET* fusion partners in *RET*-rearranged mesenchymal neoplasms. (E) A schematic representation illustrates the breakpoints and retained structural domains of *RET* fusion variants.

To determine whether MYH10::RET and CCDC6::RET have different kinase activities, FLAG-tagged MYH10::RET and CCDC6::RET were immunoprecipitated from lysates of cells stably expressing these fusion proteins. The phosphorylation status of rearranged RET was assessed using a phospho-RET (Tyr905) antibody. Both MYH10::RET and CCDC6::RET are constitutively phosphorylated and activated. Notably, the phosphorylation level of MYH10::RET was significantly higher than that of CCDC6::RET. We further evaluated the phosphorylation of MAPK, a key effector of the RET signaling pathway. Both MYH10::RET- and CCDC6::RET-expressing cells had significantly higher MAPK phosphorylation than control cells. Moreover, MAPK phosphorylation was higher in MYH10::RET-expressing cells than that in CCDC6::RET-expressing cells (Figure 5A). Lastly, an *in vitro* kinase assay was conducted to compare the kinase activities of MYH10::RET and CCDC6::RET, utilizing the IGF peptide, which is a known substrate of RET kinase. The results demonstrated that the kinase activity of MYH10::RET was approximately three-fold higher than that of CCDC6::RET (Figure 5B and C).

## 4. Discussion

We report a case of metastatic undifferentiated spindle cell sarcoma initially driven by an *MYH10::RET* rearrangement, which subsequently acquired an *NTRK* rearrangement as a mechanism of resistance to RET inhibitor therapy. Further genomic evolution has led to the emergence of kinase domain mutations in both RET and NTRK following treatment with combined RET and NTRK inhibitors. Ultimately, disease control was achieved using the next-generation tyrosine kinase inhibitor, repotrectinib. This case highlights the critical importance of sequential molecular profiling for monitoring tumor evolution and guiding precision therapy.

Spindle cell tumors with *RET* rearrangements present a wide clinical spectrum that ranges from benign lesions to aggressive high-grade sarcomas. The underlying mechanisms driving this variability in clinical behavior remain unclear. To date, a limited number of *RET* fusion partners have been reported in *RET*-rearranged mesenchymal neoplasms (Figure 5D and E).^14^ ^,^ ^49^ We hypothesized that differences in fusion partners would contribute to varying transformation capabilities. In this study, we established isogenic cell lines stably expressing MYH10::RET and CCDC6::RET, which are associated with metastatic tumors and benign LPF-NTs, respectively. Cellular assays revealed that MYH10::RET-expressing cells showed significantly faster growth, increased migration, and greater invasiveness than cells expressing *CCDC6::RET*. These observations closely mirrored the clinical behaviors associated with these fusion proteins. Further analysis confirmed that MYH10::RET had substantially higher kinase activity than CCDC6::RET. These findings suggest that different fusion partners may contribute to distinct oncogenic potentials through differential kinase activities.

This study has limitations in that it did not address the endogenous expression levels of fusion proteins in tumor cells. Expression can be regulated at multiple levels, including mRNA expression, which is influenced by various promoters associated with different fusion partners. At the protein level, factors such as post-translational modifications, protein folding, and degradation pathways can affect chimeric protein stability and function. These variations may also affect the transformation activities and clinical outcomes.

RET inhibitors are increasingly used for the treatment of RET-altered cancers. However, similar to many targeted therapies, resistance to RET inhibitors can develop over time, and the resistance mechanisms may include the acquisition of secondary *RET* mutations, activation of bypass signaling pathways (e.g., EGFR and MET), histologic transformation, pharmacokinetic factors, epigenetic alterations, and modulation of the tumor microenvironment, all of which allow tumor cells to evade targeted therapy.^50^ Our patient, with the *MYH10::RET* rearrangement, initially responded to RET inhibitors Pralsetinib and Selpercatinib, but later relapsed due to the emergence of a new *NTRK1* rearrangement. The patient then responded to combination therapy with the anti-NTRK drug entrectinib and the anti-RET drug selpercatinib, only to relapse again due to drug-resistant mutations in the kinase domains of both NTRK1 and RET.^51^ ^,^ ^52^ When resistance mutations occur in both *RET* and *NTRK1* kinase domains, the mutation with the strongest impact on drug binding typically plays a decisive role in determining resistance. The choice of a new targeted therapy depends on the specific mutation profiles and their impact on available inhibitors. Mutation abundance (variant allele frequency, VAF) can provide important clues for determining which resistance mutation is dominant. If *RET* G810C/S/R is dominant, targeted therapy can be switched to next-generation RET inhibitors, such as TPX-0046 or LOXO-260. If *NTRK1* G595R/G667C is dominant, targeted therapy can be switched to second-generation *TRK* inhibitors, such as repotrectinib (TPX-0005), which is designed to overcome *TRK* resistance mutations. Our patient subsequently responded to repotrectinib and has remained stable since then.

Our patient experienced a pralsetinib-related pulmonary infection, a condition that can be easily mistaken for tumor progression on imaging. Pralsetinib (previously known as BLU-667) is a highly effective and selective RET kinase inhibitor developed for treating RET-fusion-positive cancers, such as non-small cell lung cancer (NSCLC) and thyroid cancer. Despite its notable clinical benefits in RET-driven malignancies, Pralsetinib is also linked to several adverse effects. According to data from the ARROW clinical trial, the most common adverse events include hypertension, fatigue, gastrointestinal disturbances, and hematological toxicities. Pulmonary toxicities were reported in 12% of patients, with most cases being of grade 1 or 2 severity, whereas severe pneumonia (grade 3 or 4) occurred in only 2% of patients, sometimes necessitating dose interruptions or discontinuation.^53^ Pulmonary infections associated with pralsetinib can closely mimic tumor progression, highlighting the need for careful diagnostic differentiation. Microbiome NGS may help distinguish infections from disease progression. In our case, the patient was diagnosed with cytomegalovirus infection, which showed improvement following appropriate treatment. The precise mechanisms underlying pralsetinib-induced pneumonia remain unclear but may involve direct pulmonary toxicity, immune-mediated reactions, or interactions with concomitant therapies and underlying lung conditions. In this patient, a key consideration was the potential off-target effects of pralsetinib, which may increase susceptibility to infections or trigger autoimmune-like reactions in the lung tissue.

It should be noted that our functional comparisons of two *RET* fusions were conducted in a single cell line (NIH/3T3), a well-established system for assessing intrinsic RET activity. While this approach provides a controlled background and valuable initial insights, it does not capture the full spectrum of *RET* fusion partners or the disease-specific cellular contexts in which they operate. Future studies incorporating additional *RET* fusions and more physiologically relevant human or tumor-derived models will help to further validate and expand these findings.

## 5. Conclusion

In conclusion, we have characterized a rare metastatic spindle cell tumor harboring the *MYH10::RET* fusion. The aggressive behavior of cells expressing MYH10::RET was confirmed using isogenic cell models. Our findings suggest that different RET fusion partners of *RET* contribute to the clinical heterogeneity commonly observed in this group of tumors. Furthermore, this study highlights the critical importance of continuous genomic monitoring to identify emerging drug resistance mechanisms and tailor-targeted therapies using appropriate inhibitors.

## Data Availability

The original contributions presented in the study are included in the article/supplementary material, further inquiries can be directed to the corresponding author/s.
